# Editorial: Interaction between genes and the environment in skin aging

**DOI:** 10.3389/fragi.2025.1671721

**Published:** 2025-08-14

**Authors:** Xiaolei Ding, Sigrid Le Clerc

**Affiliations:** ^1^ Shanghai Engineering Research Center of Organ Repair, School of Medicine, Shanghai University, Shanghai, China; ^2^ Laboratoire Génomique, Bioinformatique, et Chimie Moléculaire, EA7528, Conservatoire National des Arts et Métiers, Paris, France

**Keywords:** skin, aging, environment, interaction, genetics, epigenetics (DNA methylation), microbiome

## Introduction

The skin constitutes the largest and most externally exposed organ of the human body, rendering it continuously shaped by the interplay between intrinsic genetic factors and extrinsic environmental stimuli (Tobin, 2017; Furman et al., 2025). Skin aging is associated with tissue atrophy, fragility, dryness, and loss of elasticity, clinically characterized by wrinkles, sagging, and lentigines (Ding et al., 2021; Krutmann et al., 2021). To date, genetic and environmental factors involved in skin aging have been intensively investigated, but their interaction remains poorly understood (Ng and Chew, 2022). The gene–environment interplay could be explored across complementary approaches from genetic association to RNA expression profiling, epigenetic mapping, microbiome characterization (Krutmann et al., 2021; Ratanapokasatit et al., 2022; Pozzo et al., 2024). Integrative approaches will be essential to unravel the full complexity of gene–environment interaction in skin aging and potentially develop new prevention and intervention strategies for skin aging and dermatological disorders.

This Research Topic, *Interaction Between Genes and the Environment in Skin Aging*, aims to collect and discuss complementary approaches, spanning from molecular analysis to tissue-level phenotypic assessment, to improve knowledge about how environmental factors interact with intrinsic factors in skin aging. The four original research articles included in this Research Topic offer complementary perspectives on the complexity of gene–environment interplay in skin aging and underscore the need for integrative approaches to disentangle causal pathways.

Changes in DNA methylation are recognized as a hallmark of aging in human skin, with altered methylation landscapes contributing to transcriptional dysregulation and impaired tissue homeostasis, and known to interact with environmental factors (Vandiver et al., 2015; Koenigsberg et al., 2023; Grönniger et al., 2024). Within this context, two studies, Bienkowska et al. and Falckenhayn et al., focused on the role of DNA methylation in skin aging. Bienkowska et al. developed a second-generation epigenetic age clock focusing on the correlation between methylation pattern in skin tissue and skin aging indicators such as wrinkle grade and visual facial age. The resulting clocks successfully predicted the different skin aging phenotypes, highlighting the potential of DNA methylation as a reliable predictor of skin aging traits. This work illustrates how methylation signatures can be used not only to estimate biological age but also to evaluate specific phenotypes such as visual age progression. In parallel, Falckenhayn et al. performed a screening of DNA methylation inhibitors, and tested their capacity to restore methylation patterns in skin to identify anti-aging compounds. Dihydromyricetin, a natural flavonoid identified through the screening, involved a moderate global hypomethylation in cultured keratinocytes, a reduction in the biological age of the cells, and a partial restoration of younger methylation and gene expression patterns. The study suggested that small-molecule epigenetic modulators may offer therapeutic strategies for restoring skin integrity. Together, these two contributions support a key role of methylation in skin aging and highlight the need to integrate methylation in gene-environment interaction studies.

Skin microbiome is at the interface of gene-environment interaction and plays a significant role in skin aging (Ratanapokasatit et al., 2022). The knowledge about skin aging microbiome is evolving and studies are needed to provide insights into future therapeutic options. In this context, Myers et al. investigated the relation between skin microbiota composition and aging-associated phenotypes. Through the analysis of diverse human cohorts, the authors showed an association between microbiome diversity and transepidermal water loss, age, crow’s feet wrinkles and corneometer measures. These associations suggest that the cutaneous microbiome may contribute to aging phenotypes. Importantly, the dynamic and environmentally responsive nature of the microbiome makes it a powerful model for exploring gene–environment interactions, positioning microbiota modulation as a promising strategy in the context of age-associated skin aging.

The final contribution by Obry et al. explored the interaction between lifetime sun exposure and genetic variants involved in perceived facial aging progression (PFAP). The authors used a two-step gene-environment interaction approach, using a genome-wide association study (GWAS) on PFAP to identify candidate SNPs for subsequent interaction analyses. The analysis identified significant SNPs associated with PFAP located in or near the *CGGBP1*, *PGM5-AS1*, and *CSMD1* genes. In addition, one SNP near *VANGL1* showed a significant interaction with sun exposure for perceived facial aging. These results support a putative role for telomere maintenance and keratinization in facial aging. This study underscores the potential of incorporating environmental variables into genetic analyses and emphasizes the relevance of complementary data such as methylome and transcriptome data in the interpretation of gene-environment interaction results.

The studies presented in this Research Topic offer complementary insights into how genetic and environmental factors jointly shape skin aging. By addressing this question across different biological layers, from epigenetic regulation and microbiome composition to gene–environment interactions at the genomic level, these contributions emphasize the importance of considering environmental variables in the molecular analysis of aging. The partial restoration of DNA methylation pattern, the environmentally responsive nature of the skin microbiome, and the identification of genetic variants interacting with sun exposure, all underscore the relevance of gene–environment exploration for understanding aging trajectories.

A shared message across these studies is: skin aging is a regulated and context-dependent biological process shaped by genetic predisposition, environmental experience, and their molecular intersection and it appears as a modifiable process. This view aligns with recent advances in aging biology emphasizing the role of niche-specific stem cell function, chromatin accessibility, and environmental sensing pathways in tissue maintenance (Kroemer et al., 2025). As proposed by Krutmann and colleagues, the concept of the skin exposome, the totality of environmental exposures acting on the skin, provides a valuable lens for understanding how external factors become biologically embedded (Krutmann et al., 2021).

Advancing this field will require longitudinal, multi-omic studies capable of resolving the spatiotemporal dynamics of aging at single-cell and tissue-level resolution. This integration will be critical for elucidating how aging progresses within anatomically and functionally distinct skin compartments, and how this process is modulated by modifiable environmental exposures (Solé-Boldo et al., 2020; Zou et al., 2021). These tools will not only enhance mechanistic understanding of skin aging but also enable the development of individualized, data-driven interventions.

## Conclusion

This Research Topic highlights the complementarity of genetic, epigenetic, microbial, and phenotypic data in exploring gene–environment interactions in skin aging. Together, these studies underscore the value of integrative approaches for identifying modifiable features of skin aging and guiding future research.
